# Revisiting the Proposition of Binding Pockets and Bioactive Poses for GSK-3β Allosteric Modulators Addressed to Neurodegenerative Diseases

**DOI:** 10.3390/ijms22158252

**Published:** 2021-07-31

**Authors:** Guilherme M. Silva, Rosivaldo S. Borges, Kelton L. B. Santos, Leonardo B. Federico, Isaque A. G. Francischini, Suzane Q. Gomes, Mariana P. Barcelos, Rai C. Silva, Cleydson B. R. Santos, Carlos H. T. P. Silva

**Affiliations:** 1 Departamento de Química, Faculdade de Filosofia, Ciências e Letras de Ribeirão Preto, Universidade de São Paulo, Ribeirão Preto 14040-901, SP, Brazil; suzane.quintana@usp.br (S.Q.G.); raics@usp.br (R.C.S.); tomich@fcfrp.usp.br (C.H.T.P.S.); 2 Programa de Pós-Graduação em Química Medicinal e Modelagem Molecular, Instituto de Ciências da Saúde, Universidade Federal do Pará, Belém 66075-110, PA, Brazil; keltonbelem@hotmail.com (K.L.B.S.); breno@unifap.br (C.B.R.S.); 3 Laboratório de Modelagem em Química Computacional, Departamento de Ciências Biológicas e da Saúde, Universidade Federal do Amapá, Macapá 68902-280, AP, Brazil; 4 Laboratório Computacional de Química Farmacêutica, Faculdade de Ciências Farmacêuticas de Ribeirão Preto, Universidade de São Paulo, Av. do Café, s/n, Ribeirão Preto 14040-903, SP, Brazil; leonardobrunofederico@gmail.com (L.B.F.); isaque.francischini@usp.br (I.A.G.F.); mpbarcelos@usp.br (M.P.B.)

**Keywords:** neurodegenerative diseases, GSK-3β, allosteric modulators, computer-aided drug design, cavity detection, binding pose, shape similarity, docking

## Abstract

Glycogen synthase kinase-3 beta (GSK-3β) is an enzyme pertinently linked to neurodegenerative diseases since it is associated with the regulation of key neuropathological features in the central nervous system. Among the different kinds of inhibitors of this kinase, the allosteric ones stand out due to their selective and subtle modulation, lowering the chance of producing side effects. The mechanism of GSK-3β allosteric modulators may be considered still vague in terms of elucidating a well-defined binding pocket and a bioactive pose for them. In this context, we propose to reinvestigate and reinforce such knowledge by the application of an extensive set of in silico methodologies, such as cavity detection, ligand 3D shape analysis and docking (with robust validation of corresponding protocols), and molecular dynamics. The results here obtained were consensually consistent in furnishing new structural data, in particular by providing a solid bioactive pose of one of the most representative GSK-3β allosteric modulators. We further applied this to the prospect for new compounds by ligand-based virtual screening and analyzed the potential of the two obtained virtual hits by quantum chemical calculations. All potential hits achieved will be subsequently tested by in vitro assays in order to validate our approaches as well as to unveil novel chemical entities as GSK-3β allosteric modulators.

## 1. Introduction

Neurodegenerative diseases are pathologies that affect the central nervous system (CNS), comprising medical conditions and ranging from common migraines to more serious and difficult-to-treat pathologies, such as Parkinson’s disease (PD) and Alzheimer’s disease (AD) [1]. With the world’s population aging, neurodegenerative diseases have become a common cause of morbidity and mortality in the elderly [2]. In 2016, the Global Burden of Disease Study estimated that there were 43.8 million people living with dementia around the world, with a total of 9 million deaths per year caused by neurodegenerative diseases [1,3].

The enzyme glycogen synthase kinase-3 (GSK-3) is a ubiquitous and highly conserved serine/threonine protein kinase, occurring in the isoforms α (alpha) and β (beta), that plays a fundamental role in the emergence and worsening of CNS and neurodegenerative diseases such as AD [4]. GSK-3β acts as phosphorylating tau proteins, which are known to stabilize the microtubules by securing tubulin assembly. In the CNS, the levels of GSK-3β are higher and, with aging, its expression tends to increase. This may, thereby, cause the accumulation of tau proteins in the cytoplasm, leading to microtubule disassembly, loss of neuronal integrity, and, eventually, neurofibrillary tangle (NFT) formation [5,6]. Additionally, recent studies have shown that overexpression of GSK-3β is associated with increased β-amyloid production, local plaque-associated microglial-mediated inflammatory responses, and memory impairment [7].

When it comes to GSK-3 regulation/modulation, one should note that there are several complex mechanisms under study due to its role in a wide range of biochemical processes and signaling pathways [8]. Worthy of mention, this kinase may be inhibited by various endogenous substrates [9], such as bivalent zinc ions [10], in addition to being actively or passively involved in different pathways, e.g., Wnt/beta-catenin, TP53, Notch, and others [11]. Moreover, GSK-3 can be related to transcriptional and/or translational mechanisms as these pathways are regulated by micro-RNAs [11,12] and also because it is capable of modulating post-translational modifications of histones [13], for instance. Nevertheless, we emphasize that further introduction will describe aspects concerning the development of small-molecule inhibitors and their related GSK-3β modulation.

One of the most studied GSK-3β inhibitors is lithium, which is used as a pharmacological alternative to treat psychiatric disorders such as bipolar disorder as well as neurodegenerative diseases (e.g., AD and PD) [14]. Lithium is a traditional and noncompetitive inhibitor of the enzyme that does not hinder the binding of the substrate (adenosine triphosphate, ATP) and other protein residues to be phosphorylated within the catalytic (orthosteric) site [15]. The most accepted hypothesis of its mechanism of action is that lithium competes with magnesium in a GSK-3β site sensitive to lithium, preventing the magnesium from binding. As a consequence, the ATP conformation is destabilized in the catalytic cavity, preventing it from being cleaved and, therefore, inhibiting enzyme activity [15,16].

Also commonly reported is the class of GSK-3β inhibitors known as ATP competitive inhibitors, which compete for the same site as ATP in the enzyme’s orthosteric cavity, and from which compounds with considerable bioactivity are known (such as indirubin and hymeldiasine, among others) [17,18,19].

Non-ATP competitive inhibitors—which generally include substrate-competitive and allosteric inhibitors—have been studied as an alternative to ATP-competitive inhibitors since they potentially cause fewer side effects, despite presenting lower potency/affinity towards GSK-3β. Within this class, we can highlight tideglusib, which inhibits GSK-3β irreversibly but does not block the whole pool of enzymes within cells; it has reached phase II clinical trials [19,20]. Nevertheless, commonly and unfortunately, there is no further elucidation on how (and by which mechanism) non-ATP-competitive compounds exert inhibition, and there is also a lack of information concerning which cavities (or pockets) of GSK-3β they act on.

**Figure 1:**
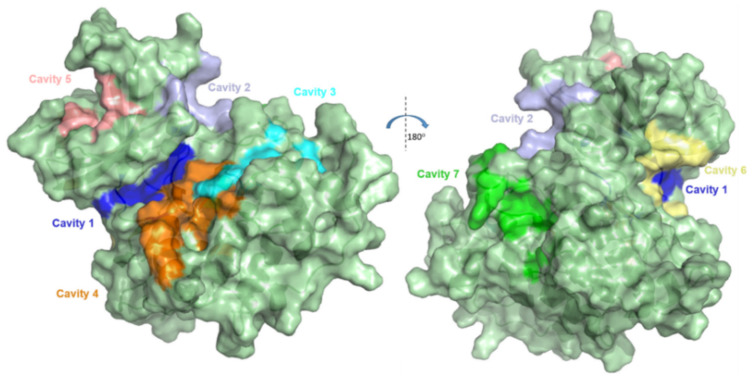
Representation of the 7 cavities/pockets present in GSK-3β. Image prepared using PDB ID 1PYX in Pymol [22]. Adapted with permission from Palomo et al. *J. Med. Chem*. **2011**, *54*, 8461–8470 [21]. Copyright 2021 American Chemical Society.

Palomo et al. [21] applied a computational methodology to map potential and common pockets found on the surface of GSK-3β by means of fpocket software, thus describing the presence of 7 different well-maintained (conserved) cavities. Such pockets were numbered and classified as follows: (1) ATP active site; (2) substrate site; (3) axin/fratide binding site; (4) allosteric site; (5) allosteric site; (6) allosteric site; (7) allosteric site. These last four were highly indicated as potential allosteric sites, as depicted in Figure 1. This data should be useful to track non-ATP-competitive inhibitors’ mechanisms and also to establish a distinction as to when they could, indeed, act as allosteric inhibitors.

**Figure 2:**
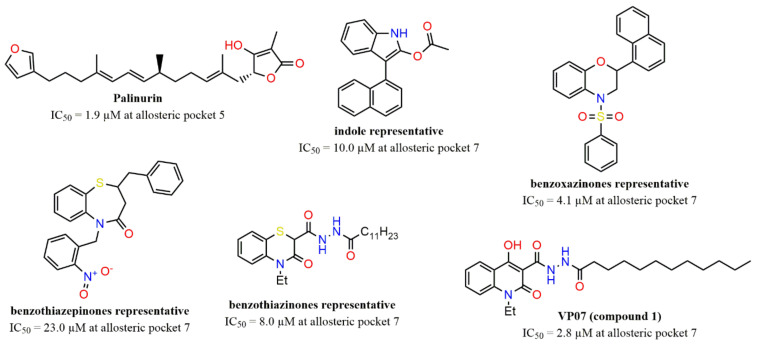
Representation of different classes of GSK-3β allosteric inhibitors potentially binding to corresponding pockets.

According to Silva et al. [19], there are few classes of GSK-3β allosteric inhibitors discovered so far, and there are still some peculiarities of respective mechanisms to be unveiled. For instance, palinurin, a compound found in marine organisms, was revealed as a new class of GSK-3β allosteric inhibitors, with IC_50_ = 1.9 μM, along with suggestions that it should bind to allosteric Pocket 5 (from Figure 1) [23]. Other studies have also revealed further classes of GSK-3β allosteric modulators that would possibly bind to Pocket 7, such as benzoxazinones [24], indoles [24], benzothiazepinones [25], and benzothiazinones [26,27]. These classes of scaffolds are depicted by representative compounds in Figure 2.

Regardless, one should note that allosteric inhibition of this enzyme clearly points to potential advantages, such as higher selectivity and mild-to-high potency, minimizing side effects overall due to ligands from different classes being able to bind at specific cavities [23,28]. Moreover, it is worth mentioning that overinhibition of GSK-3β might be disfavorable due to the activation of Wnt signaling, and, thus, allosteric modulation appears to be a mild option for the optimal inhibition level of this enzyme [24,29].

Another important study [21,28] reported the discovery of the class of quinolonic derivatives as important allosteric modulators of GSK-3β through interaction with allosteric Pocket 7. Among this class, compound **1** (VP07) stands out (see Figure 2), with IC_50_ = 2.8 µM, measured by in vitro enzyme activity assays. It is worth mentioning that the compound **1** inhibition mechanism was unequivocally described as allosteric after the execution of kinetic studies employing double-reciprocal plots, in which the ATP/substrate concentration is maintained and the substrate/ATP/inhibitor concentrations are varied.

Furthermore, compound **1** has presented promising neuroprotective properties and therapeutic potential, as revealed by tests in human samples from patients with congenital myotonic dystrophy type 1 and spinal muscular atrophy. In addition, other analogs of **1** were also designed and tested, e.g., compounds **18** and **24,** which were also able to show good activity towards the allosteric modulation of GSK-3β [28]. As for the interaction mode of compound **1** with allosteric Pocket 7, this was described using only a blind-docking procedure—a docking simulation in which the entire surface of the protein is considered in order to assess which cavity the ligand presents greater affinity towards—using only one docking software.

In this way, it would be extremely convenient to deepen these studies concerning the allosteric cavities of GSK-3β, i.e., to reinforce the proposition of possible allosteric pockets as well as to evaluate the bioactive poses of allosteric inhibitors towards the binding pockets of such enzymes. Moreover, one should take into account the important aspect that there is no crystallographic complex of this enzyme structure, with allosteric ligands, deposited in Protein Data Bank (PDB, https://www.rcsb.org/, accessed on 18 May 2021) yet.

Therefore, the objective of this work is to apply a diverse and robust set of methodologies to reinvestigate, and maybe reinforce, the allosteric mechanism suggested [21,28], especially regarding which of the four allosteric cavities previously described has indeed greater druggability with compound **1** (i.e., which binding pocket has greater ability to interact with it). Moreover, we further evaluate its most likely corresponding mode/pose of interaction (i.e., bioactive pose). Our methodologies consist of cavity detection, ligand 3D-shape analysis and docking (with the validation of corresponding protocols/queries), and molecular dynamics (MD); thus, we are able to provide new structural data that should expand the knowledge of GSK-3β allosteric inhibition. We further used the best proposed bioactive pose of **1** for the prospect of new allosteric modulators by ligand-based virtual screening (LBVS) and analyzed the potential of the two representative virtual hits by quantum chemical calculations. These two, plus other virtual hits, will be tested by in vitro assays, soon to be published. Finally, we expect that the results provided in this work will contribute to the development of new allosteric modulators of GSK-3β, which is a relevant kinase to be used as a target to treat neurodegenerative diseases.

## 2. Results

### 2.1. Detection and Prediction of the Potential of Allosteric Pockets

**Figure 3:**
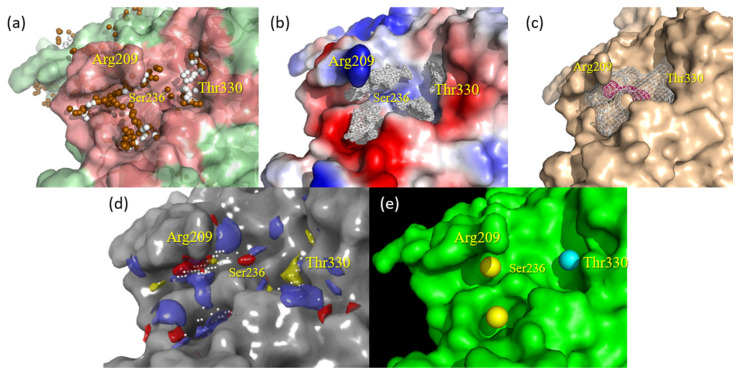
(**a**) Cavity detected using fpocket, represented here by brown (polar groups) and white (apolar groups) spheres. (**b**) White mesh shape of cavity predicted by Superstar. (**c**) Best scoring pocket by metaPocket, represented by white mesh (cluster of sites indicated in consensus by three methodologies) and pink spheres (mass center of the global site). (**d**) Sitemap cavity occupied by white spheres. (**e**) Cavity with the highest potential of representing an allosteric site, according to PARS. All images were produced using Pymol [22], showing Pocket 7 of GSK-3β (1PYX.pdb).

In order to explore and detect the pockets present on the GSK-3β surface, five different software/webservers—also commonly classified as “binding site predictors” or “ligand-binding cavity detectors”—were employed, i.e., fpocket [30], Superstar [31], metaPocket [32], Sitemap [33], and PARS [34]. In general, these software exert pocket detection algorithms, which allow the visualization of each cavity shape/volume along with corresponding calculated “scores” values, according to the potential of the cavity to interact with potential ligands and/or drugs.

fpocket and Superstar allow the visualization of the shape of the pockets, apparently indicating a more compatible format of allosteric Cavity 7 with compound **1** (see Figure 3a,b). fpocket, in addition to well-defining the pocket compatible with compound **1**, provided feature spheres that characterize the cavity occupancy by polar groups (brown) and nonpolar groups (white), partially indicating a preference for the apolar/hydrophobic chain of **1** to accommodate close to the Thr330 residue and corresponding hydrophobic pocket.

metaPocket mapped Cavity 7 in consensus by means of three different methodologies (LigsiteCS, fpocket, and Ghecom), indicating both the pocket clustered by these three, considering the respective spatial similarities (in blank mesh) as well as the center mass of such clustered pocket (in pink spheres), indicating the nearby residues with the greatest potential for interaction (see Figure 3c). Furthermore, metaPocket provided a z-score for Cavity 7, with a value of 3.25 (only smaller than the z-score value of the catalytic site, 22.94), thus indicating its high propensity to act as an interaction/binding site.

Sitemap indicated the regions that best define the mapping of Cavity 7 (Figure 3d), indicated by white spheres, in addition to assigning the second-highest score of 0.898 (also only lower than the 1.021 catalytic site) for such allosteric cavity. The other colors mapped by this software indicate the pharmacophoric patterns of the site, which will be discussed later (see Section 2.3).

The software PARS assesses the volume, flexibility, and structural conservation of the amino acid residues that constitute the cavity of interest. It was able to indicate three independent spheres that make up Cavity 7 (Figure 3e). The first ranked yellow sphere underneath Arg209 was best scored as the putative allosteric site, i.e., considered the largest one, with a *p*-value of 0.71 and structural conservation of 12.60%. The secondly ranked yellow sphere showed a *p*-value of 0.79 and 25.80% of conservation, while the third light blue sphere presented a *p*-value of 0.47 and 56.90% of conservation. The three of them together, especially the first and third spheres, should globally indicate that Pocket 7 represents the most promising allosteric site in comparison to the whole structure and further cavities present on the GSK-3β surface.

### 2.2. Docking Assessment—Pocket Perspective

A preliminary docking assessment was carried out in order to detect which GSK-3β allosteric pocket should present greater accommodation and binding affinity with compound **1**. For this, GOLD [35,36], Glide [37,38], AutoDock [39,40], and FRED [41,42] software were employed to run docking simulations of **1**, individually, in each of the four allosteric cavities (from Figure 1), and the respective score values were compared.

**Figure 4:**
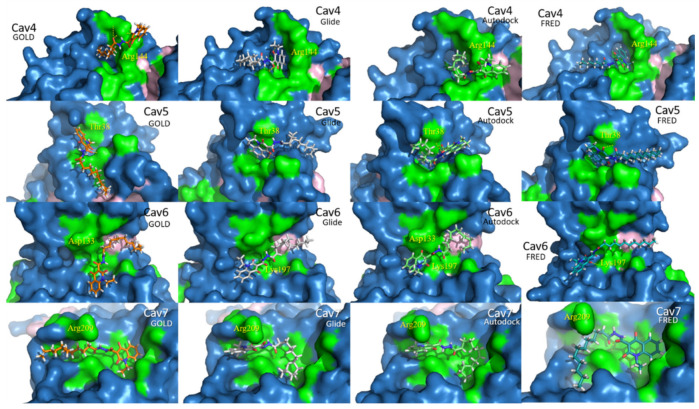
Representation of the docking poses obtained for compound **1** within each allosteric cavity (4, 5, 6, and 7) of protein GSK-3β (1PYX.pdb) using GOLD, Glide, Autodock, and FRED software. Image prepared in Pymol [22].

In consensus, the four docking software indicated a more favorable score value for **1** docked in Cavity 7 than in the other three allosteric pockets, as can be seen in Table 1. However, in Figure 4, one may observe that the best punctuated poses, obtained by each software, do not show homogeneity in relation to their poses. In other words, the docking pose of **1** in Cavity 7, obtained using GOLD and FRED, accommodated the hydrophobic chain under Arg209, while Glide and Autodock accommodated the quinolonic ring in this same region.

Therefore, based on the results observed so far, it is not possible to conclude which is the most representative pose (supposed bioactive pose) of **1** in Pocket 7. For this reason, in the next section, we describe the application of other complementary computational methodology to clarify this.

### 2.3. Evaluation of Compound 1 Pose within Allosteric Pocket 7

Three types of software were used to map contour/surface areas as well as generate pharmacophoric hotspots, with the purpose of clarifying the visualization of the pose of compound **1** in a coherent way (i.e., taking into account its structural and physicochemical characteristics) against allosteric Cavity 7 of GSK-3β. This complementary step was necessary, considering that the preliminary docking study (Section 2.2) indicated different poses (in terms of conformation and orientation) for **1** in Cavity 7.

**Figure 5:**
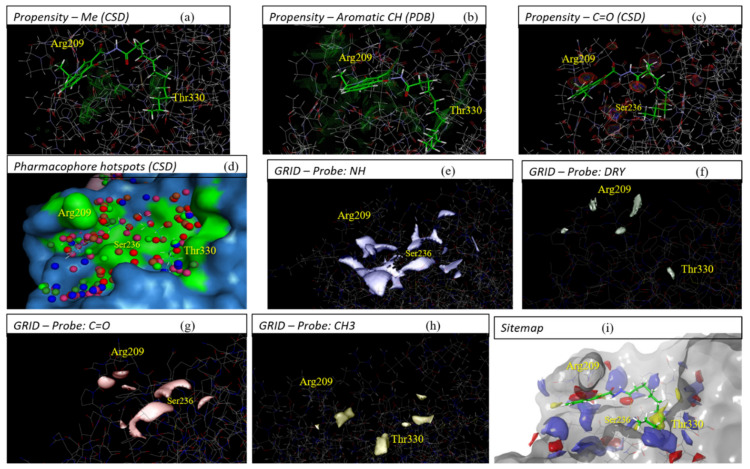
(**a**–**c**) Contour maps generated with Superstar based on CSD/PDB data, using propensity function and the respective probes; red dots indicate a chance 2× higher of the probe being found in that position than randomness; green 4× and blue 8×. (**d**) Pharmacophore hotspots generated by Superstar considering the CSD database; spheres in green (aliphatic or aromatic C-H), blue (nitrogen, N), and red (oxygen, O). (**e**–**h**) Surface maps generated by GRID using the respective probes in different ranges of energy interactions. (**i**) Surface maps generated by Sitemap; maps in yellow (hydrophobic), red (hydrogen bond acceptor), and blue (hydrogen bond donor). All images show Pocket 7 of GSK-3β (1PYX.pdb); where possible, compound **1** (Glide pose) was superimposed for analysis.

The first software used here was Superstar [31], which generated contour maps and pharmacophoric hotspots, allowing us to infer that: the aliphatic C chain of compound **1** must be favorable close to residues Arg328, Thr330, and Pro331 (Figure 5a,d); the presence of a group C=O just above the N-H of Ser236 is quite likely (Figure 5c), and aromatic C are likely under Arg209 (Figure 5b).

Similar to Superstar, the software GRID [43] generated contour maps (by means of molecular interaction fields (MIFs)) that showed possible interactions with the probes applied. It was possible to strongly deduce that the aliphatic C chain tends to be close to the Thr330 residue (Figure 5f,h), N-H below Arg209 (Figure 5e), and C=O just above the N-H of Ser236 (Figure 5g).

Finally, Sitemap [33] indicated the same interactions mentioned above for the two software, in addition to confirming that the aliphatic C chain of **1** should be better accommodated in the hydrophobic pocket of Cavity 7, which consists of residues Arg328, Thr330, and Pro331 (Figure 5i).

### 2.4. Refinement and Validation of Docking Protocols—Pose Perspective

The results obtained using the four docking software previously mentioned revealed that AutoDock and Glide generated poses for **1** in agreement with contour map evaluations, while GOLD and FRED generated poses with inverted orientations within allosteric Cavity 7 of GSK-3β. Thus, in order to obtain an even more reliable pose of **1** (in view of the different docking methodologies as well as the other previously used methodologies), a docking assessment was carried out to clarify the ‘pose issue’ in two steps. First, different protocols were evaluated for each software through refinement and variations of the parameters/settings to run docking simulations. In a second step, validation of the best protocols was performed for each software using a set of 40 compounds (**1** and its analogs, with known IC_50_ values against the allosteric inhibition of GSK-3β [28]; 27 actives and 13 inactives; threshold of 20 µM).

Each protocol (that is, each adjustment of the parameters used in each docking simulation) was initially tested for analysis by obtaining the pose of compound **1** in Cavity 7. Then, they were expanded to evaluate the poses obtained for the 40-molecules dataset. In this way, the most significant/efficient protocols of each software were selected during the second stage, in which a validation by ROC (receiver operating characteristic) curves was carried out to evaluate the protocol with greater capacity to rank active/inactive compounds among the set of 40 analogs (see Tables S1 and S4).

After evaluating different protocols for both GOLD and Glide, the best ones were selected and plotted as ROC curves (see Figure S1) along with the docking results obtained by the unique protocols applied for Autodock and FRED. It is observed that among the four evaluated software, Glide presented a higher AUC (area under the curve) value, that is, a greater capacity to distinguish and classify the active and inactive compounds contained in the set of 40 analogs of **1**. Thus, in this previous validation, Glide was considered the software with the best performance to predict poses and assign the respective score values in docking simulations, considering the limited set of compounds that was used (we try to overcome this issue below—see Section 2.5).

### 2.5. Optimizing Docking Validation

In order to optimize docking validation, especially concerning the use of a larger dataset, with known allosteric inhibitors of GSK-3β, we expanded the dataset to 88 compounds (including 37 actives and 51 inactives), considering an IC_50_ threshold value of 20 µM to sort them into active/inactive. Table S1 shows the dataset of 88 GSK-3K allosteric inhibitors, compiled from the literature, and their respective IC_50_ values.

In addition, we performed docking validation with the addition of decoys generated by the webserver DUD-E [44], considering the structure of 37 active compounds, which resulted in ca. 50 decoys per active compound.

**Figure 6:**
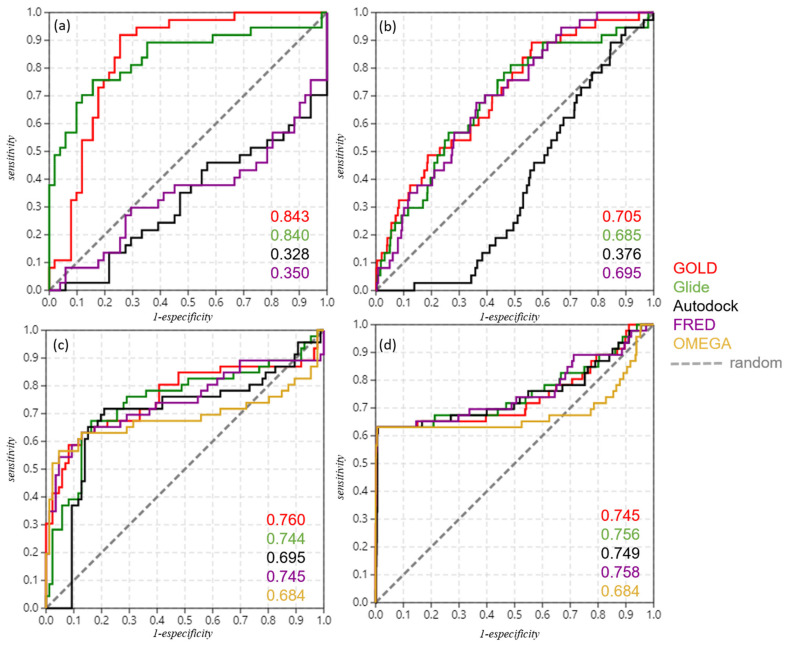
Docking validation by ROC curves and the respective AUC values obtained by each software using (**a**) dataset of 88 compounds and (**b**) 88 compounds plus decoys. vROCS validation by ROC curves and the respective AUC values obtained by each query of corresponding software using (**c**) dataset of 88 compounds and (**d**) 88 compounds plus decoys.

The same procedures for the validation of the best docking protocols for each software, using the restricted dataset of 40 compounds, as previously presented in Section 2.4, were applied here for this new set of 88 compounds, with and without the decoys generated by DUD-E. In this manner, the ROC curves were plotted, and the corresponding AUC values were obtained (as shown in Table 2 and Figure 6, as well as in Table S4 with other relevant metrics).

Table 2 shows that when considering only the 88-compounds dataset, GOLD and Glide showed similar and equivalent AUC values, next to 0.8, and Autodock and FRED were also equivalent but with AUC values next to 0.3. A similar observation can also be made regarding the AUC values obtained with the 88-compounds dataset plus the decoys generated. This demonstrates that GOLD and Glide show better performance in actives ranking, even when the structural diversity of the compounds was expanded for the set of 88 compounds (and even with decoy addition). In particular, FRED similarly showed improvement in its performance (according to its AUC values, which rose from 0.350 to 0.695) when applied to the set of 88 compounds plus decoys.

In general, one should note that GOLD and Glide showed equivalent performance in both docking and vROCS validations (see also metrics in Table S4). Nevertheless, taking into account that Glide corroborates previous pose prediction studies (cavity detection, surface mapping, and the literature [21]), whereas GOLD has not, leads us to infer that Glide’s results were more reliable among the evaluated software.

### 2.6. Shape Similarity and Query Validation

In order to validate different queries to be used in a 3D shape similarity search by ROCS [45] (or vROCS [46]), the four best docking poses of compound **1**, generated by each docking software (GOLD, Glide, Autodock and FRED), and one energy-minimized conformation generated by OMEGA [47] were submitted as queries. Two datasets consisting of 88 compounds and/or 88 compounds added with decoys were used. It is worth noting that such datasets were previously submitted to max 300 conformers per molecule using OMEGA [47].

We thus proceeded with such validation in a similar way as docking validation, i.e., using ROC curves and the corresponding AUC values. Figure 6 and Table 2 (also Table S4) show, respectively, the ROC curves and AUC values obtained using each query.

**Figure 7:**
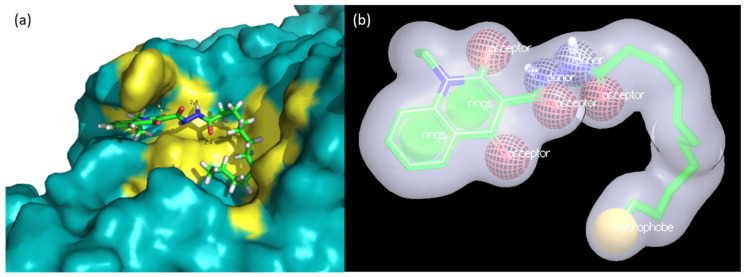
(**a**) Well classified and reliable pose of **1** within allosteric Cavity 7 of GSK-3β (1PYX.pdb), obtained by Glide docking software. Image produced in Pymol [22]. (**b**) Conformational pose of **1** presented by vROCS, showing its shape (in transparent grey) and its color chemical features (aromatic rings in green, hydrogen bond acceptors in red, hydrogen bond donors in blue, and hydrophobic groups in yellow).

One may observe that for all the queries used, the AUC values obtained were above what is considered random (AUC = 0.5). In general, they present reasonable and apparently equivalent values to rank the ‘actives’ and ‘inactives’ contained in each dataset. However, we considered that the Glide pose (see Figure 7) showed greater reliability due to the fact that, in general, it consensually showed higher AUC values for both docking and shape similarity validations in addition to corroborating with the studies of pose/pocket predictions and map surfaces (performed in Section 2.1 and Section 2.3).

### 2.7. Molecular Dynamics Study

With a view to the evaluated results obtained up to this point and aiming to further confirm the docking pose capable of representing a more reliable bioactive pose of compound **1** in allosteric Cavity 7 of GSK-3β, we conducted a molecular dynamics (MD) study. For this, we used the docking poses obtained by the Glide and GOLD software as input files in the MD simulations, considering that one is in inverted conformation to the other.

**Figure 8:**
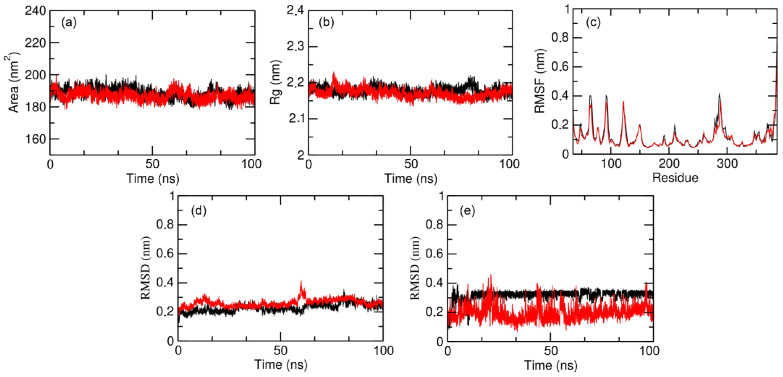
Results obtained by MD simulations carried out for the two poses of compound **1,** previously obtained with Glide (black) and GOLD (red), and the respective protein–ligand complex structures within allosteric Pocket 7 of GSK-3β. Resulting graphs showing (**a**) SASA, (**b**) Rg, (**c**) RMSF for the complexes, (**d**) RMSD for the complexes, and (**e**) RMSD only for ligands (poses).

In this way, we could obtain important information about the dynamic behavior of the compound’s poses in the function of 100 ns trajectories and in solvated medium. Such simulations allow us to analyze the stability of the complexes formed with each pose within the respective allosteric pocket, as well as the flexibility of the residues present in the enzyme. These data were achieved by obtaining results concerning solvent-accessible surface area (SASA), total radius of the gyrate of the protein in space (Rg), root-mean-square fluctuation (RMSF), and root-mean-square deviation (RMSD) for the protein complexes and RMSD only for each ligand (pose) during trajectory, as presented in Figure 8.

To determine the stability and integrity of the protein complexes during each trajectory, we evaluated their compaction using the SASA and Rg measurements, which were extracted every 2 fs and plotted in graphs (Figure 8a,b, respectively). With regard to the SASA of GSK-3β, both protein complexes remained compact during the trajectory, i.e., their secondary and tertiary structures were maintained during the entire simulation without unfolding. This information can be especially observed through corresponding values of ~190 nm^2^, which did not show significant variation. Such information from the SASA was confirmed by analyzing the graphs of Rg measurements. One can see that there were also no large variations in Rg values in both simulations, showing that the structural dimension of the complexes remained constant throughout the trajectory and, therefore, confirming that the protein complexes remained compact and stable.

Regarding the RMSF graphs (Figure 8c), one may note that the protein complexes with both poses showed four peaks next to 0.4 nm, indicating a large fluctuation in the amino acids that make up these regions. From these, the first two peaks are represented by one next to Phe67 and the other around Phe93; in fact, both residues are part of Pocket 2 from GSK-3β (see Figure 1), which corresponds to the substrate pocket. We speculate that their considerable fluctuations could indicate corresponding modulations as a consequence of the allosteric binding of compound **1** in Pocket 7. However, further data to confirm this may need to be further investigated. Moreover, from the literature, suggestions to the allosteric modulation of **1** bound to Pocket 7 were made by inferring that residue Glu211 approximates that of Lys205 [28]. In our work, we also checked a considerable RMSF peak for this first residue, which might corroborate this information.

In addition, the balance and stability of complex formation were also analyzed by evaluating the RMSD measurements along the trajectory. From Figure 8d, we can then observe that there were no large variations in the RMSD values in both simulations. Moreover, RMSD mean values for Glide and GOLD were 0.238 ± 0.031 and 0.265 ± 0.025 nm, respectively, which could mean a slight indication of better stability for the complex with **1** (pose from Glide) in GSK-3β Pocket 7.

Finally, we analyzed the RMSD values only for the ligands (poses) of each complex (Figure 8e). From this analysis, it was possible to evaluate whether the ligands remained in their initial pose (i.e., in their docking poses in terms of conformation + orientation) of the simulation throughout the trajectory or if the dynamics of the system (protein, ligand, solvent) favored their movement, leading to readjustments in their poses as the consequence of other interactions formed between their complexes with the protein. We observed that the Glide pose (black) showed constant RMSD ~0.3 Å, whereas the GOLD pose (red) showed some variations of RMSD ~0.2 Å. These RMSD values are different only by a unit of 0.1 and should not, in general, indicate a significant difference between them. On the other hand, observable variations of the GOLD pose should suggest that its positioning during the trajectory might adopt different poses, for instance, due to the movement of its long and flexible aliphatic chain.

### 2.8. Virtual Screening

Given the obtainment of our reliable predicted bioactive pose for compound **1** (using Glide, see Figure 7), we used such pose in a virtual screening (VS) campaign. For this purpose, we applied ligand-based methodology (LBVS), that is, 3D shape similarity using the software ROCS, inputting our predicted pose as a query and applying it to three selected and prepared databases with millions of commercial compounds (details in Section 4.9).

Hence, we filtered the databases with ROCS, retrieving the 5000 top-ranked molecules according to the respective ROCS *TanimotoCombo* indices (ROCSTC; shape + color) indices. Moreover, screening was carried out through 3D electrostatic similarity using EON, selecting the 1000 top-ranked EON *TanimotoCombo* indices (EONTC) from each database. See Table S2 regarding the number of compounds filtered in each step of our VS campaign.

In sequence, compounds were submitted to pharmacokinetic and toxicological (ADME/Tox) predictions using QikProp and DEREK, respectively.

Lastly, we applied our previously developed and reliable docking protocol (using Glide) in order to evaluate the ability of survival compounds to establish consistent intermolecular interactions within allosteric Pocket 7 of GSK-3β. Furthermore, visual inspection of their structures was narrowly conducted to then select the most promising compounds with structural diversity. We selected ~30 compounds to purchase and perform in vitro enzymatic assays; however, we will only reveal two structures and the corresponding properties, which, in fact, are also included in further quantum chemical calculations analysis (see Section 2.9).

**Figure 9:**
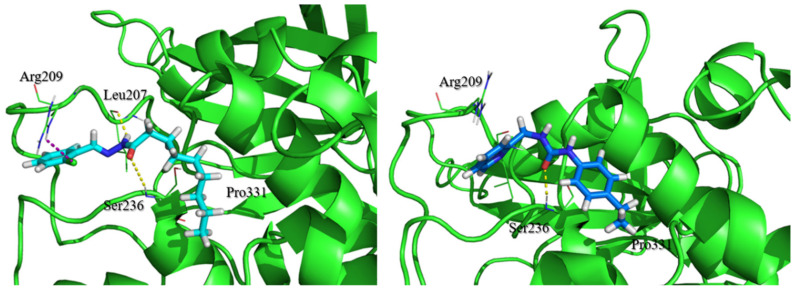
Representation of docked poses of **LCQFGS01** and **LCQFGS02** within GSK-3β (1PYX.pdb) allosteric Cavity 7, obtained by Glide-developed docking protocol. Hydrogen bonds in yellow, and halogen bonds in purple. Images produced in Pymol [22].

Compounds **LCQFGS01** and **LCQFGS02** presented structural diversity when compared to query compound **1**. Table 3 shows their ADME/Tox properties, which indicate the preferable physicochemical parameters and also a better profile of both these compounds to act as GSK-3β allosteric modulators through the CNS. In addition, Figure 9 shows docking simulations performed for both virtual hits, showing the ability of their poses to keep important interactions in GSK-3β allosteric Cavity 7 (the same observed for **1**).

### 2.9. Quantum Chemical Studies

In this section of the work, we selected five compounds to investigate the relationships between their quantum chemical features and their potential to perform intermolecular interactions within allosteric Pocket 7 of GSK-3β. The five selected compounds correspond to compounds **1**, **18**, and **24** (as three reference compounds from the literature [28] and also the most active ones (lowest values of IC_50_, see Table S1) towards GSK-3β allosteric inhibition), along with compounds **LCQFGS01** and **LCQFGS02** (as two potential hits obtained by our VS campaign in Section 2.8).

The idea is to compare the ability of these two virtual hits to perform the inhibition of GSK-3β allosterically in a similar manner to those three reference compounds in terms of the results observed from such quantum chemical calculations. It is worth emphasizing that the exact docking poses of all five compounds were kept during this study.

Following the procedures described in Section 4.10, in a preliminary evaluation to choose the most appropriate basis set for quantum chemical calculations, we observed that basis set B3LYP/6-311++G(2d,2p) released a higher mean average energy of formation than B3LYP/6-311+G(d,*p*) for the five selected compounds (see Table S3). Assuming that the greater such energy, the more stable the respective compounds, we considered that basis set B3LYP/6-311++G(2d,2p) was greatly indicated for energetic analysis in further calculations.

Moreover, we calculated the highest occupied molecular orbital (HOMO), the lowest unoccupied molecular orbital (LUMO); the GAP between LUMO and HOMO, the ionization potential (IP), and the spin density for the five compounds, as shown in Table 4.

From Table 4, one can see that all values of HOMO were superior to −6.00 eV. Compounds **18** and **LCQFGS02** presented the highest HOMO values of −6.19 and −6.10 eV, respectively, whereas **LCQFGS01** showed the lowest HOMO value of −6.59 eV. Regarding LUMO energy values, compound **1** was the lowest (−1.94 eV), followed by compounds **24** (−1.86 eV) and **18** (−1.79 eV), while **LCQFGS02** presented the highest LUMO value of −1.03 eV. In addition, the calculated GAP values—meaning the difference between LUMO and HOMO energy values—were able to show that **18** should be the most reactive compound as it presented the lowest value of 4.40 eV, followed by **1** (4.48 eV) > **24** (4.60 eV) > **LCQFGS01** (4.82 eV) > **LCQFGS02** (5.07 eV). Furthermore, compounds **1**, **24**, and **LCQFGS01** showed IP values higher than 180 kcal/mol, while **18** and **LCQFGS02** showed the lowest IP values of 174.75 and 177.12, respectively.

It is worth noting that in general terms, low GAP values are related to high reactivity and low chemical stability of compounds (and vice-versa), whereas HOMO and LUMO relate to the ability to donate and accept electrons, respectively [48,49,50]. Additionally, IP is related to nucleophilicity and the electron-donating ability of compounds [51,52]. Nevertheless, here, we extrapolate these chemical reactivity concepts laterally, interpreting them in terms of the ability of compounds to perform intermolecular interactions with the amino acid residues of GSK-3β.

**Figure 10:**
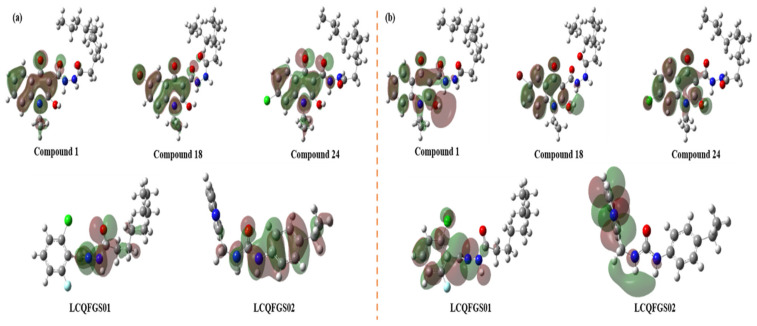
(**a**) HOMO and (**b**) LUMO of compounds **1**, **18**, **24** (most active reference GSK-3β allosteric modulators) and our two virtual hits, **LCQFGS01** and **LCQFGS02**.

Figure 10 depicts the HOMO and LUMO orbital distributions throughout the chemical structures of each of the five selected compounds. Additionally, to facilitate comprehension, Figure S2 shows their 2D color maps in terms of blue and red portions of molecules with the corresponding predominance of each frontier molecular orbital (FMO) character.

One may observe from Figure 10 that compounds **1**, **18**, and **24** have shown the HOMO homogeneously distributed on their quinolone moieties (blue portion, see Figure S2), which indicates the important role of such a group (and the attached substituents) to HOMO. Regarding the LUMO of these same compounds, they have similarly shown an overall distribution on the corresponding quinolone moieties, indicating that in contrast and parallel to HOMO results, they tend to act as electrophiles. However, the long aliphatic hydrocarbon chain (the hydrophobic part of molecules) has not been included as important for either HOMO or LUMO.

In fact, this corroborates the expected behavior of these compounds when bound to allosteric Pocket 7 of GSK-3β since the quinolone moiety is presumably interacting with Arg209 by three possible intermolecular forces: cation-π (between N^+^ from Arg209 and π-conjugated electrons from quinolone), hydrogen bond (between N-H from Arg209 and C=O in Position 4 of quinolone ring), or electrostatic/ionic. Despite the HOMO predominance on the Arg209 N terminal side chain [53], this could be allowed in view of the versatility of the kind of interaction for these three. Such corroboration has been shown by our docking studies (and the corresponding interactions) as well as in literature [28]. Moreover, for these three compounds, we see that their oxygen—from carbohydrazide C=O, next to the hydrophobic chain—possesses ready-to-interact electron pairs in accordance to the depicted HOMO orbitals, which should allow the formation of the important hydrogen bond with the N-H of Ser236.

For compounds **LCQFGS01** and **LCQFGS02**, the corresponding red portions (see Figure S2)—i.e., methylenehydrazide and carbamide moieties, respectively—played a key role to the HOMO, especially indicating that their C=O group might indeed form the important hydrogen bond with the N-H of Ser236. Besides this, their LUMOs, distributed through corresponding moieties of halogenated benzene and pyridine, respectively, make sense since they also can interact with Arg209, which shows a HOMO character as well [53]. Moreover, in the specific case of **LCQFGS01,** this analysis also corroborates the possibility of forming a halogen bond between its Cl and the N-H of Arg209 (see Figure 9).

Lastly, spin density contributions were also evaluated (see Figure S3), considering that this should measure chemical stability and that the electron donation capacity depends on their cation-free radical stability. The difference between the HOMO and spin density distribution shows each group involved in radical stabilization using numerical values. In the blue portion (see Figure S2) of compounds **1**, **18**, and **24**, there was a higher contribution in radical stabilities, with a highlight to the presence of nitrogen and oxygen atoms in the quinolonic ring and electronic distribution in their planar structures. Compound **LCQFGS01** showed greater spin density and a HOMO concentrated in its red portion, with a smaller distribution when compared to **LCQFGS02**; thus, these data, in general, corroborate the analyses made for the FMOs.

## 3. Discussion

It is worth mentioning that Palomo et al. [21] performed a comparison between several GSK-3β PDB structures using only fpocket software in order to ascertain the consensual presence of the seven cavities that were found in these different structures, from which Cavities 4, 5, 6, and 7 have shown to be allosteric in potential. Here, in a complementary way, we evaluate which one among the cavities previously described as allosteric would have the greatest potential to interact with inhibitors/ligands (especially with **c**ompound **1**).

Our approach, in this work, started by analyzing all possible (shallow or buried) cavities detected on the surface of GSK-3β. Using five different software/webservers, all of them indicated in consensus that Cavity 7 presents a higher likelihood of representing an allosteric site as well as reasonable druggability scores, usually only smaller than the actual active site of GSK-3β. Considering these results of cavity detection and prediction of their potential among the four allosteric cavities previously described, Cavity 7 is, indeed, potentially indicated as a cavity more likely to interact with a given orphan molecule, e.g., a non-ATP-competitive inhibitor of such enzymes.

From preliminary docking results (Section 2.2), **1** has shown to bind more effectively (in terms of each scoring function used in each software) when bound to allosteric Pocket 7 rather than the other three allosteric pockets. This points to the existence of higher compatibility of the physicochemical/structural features of compound **1** within such a pocket. In fact, considering that two types of software (GOLD and FRED) predicted a binding pose inverted with relation to poses predicted using the other two types of software (Glide and Autodock), this might indicate that the actual pose of **1** inside Cavity 7 could be indifferent. This pose independence, however, was further investigated in order to evaluate which docking methodology furnishes greater/more reliable pose prediction.

In addition, it is worth stressing that compound **1** presents 14 rotatable bonds and its C_11_H_23_ hydrocarbon aliphatic chain allows the structure to adopt many conformations, regardless of the conformational search algorithm employed by each docking software (Glide and FRED = systematic search, while GOLD and Autodock = stochastic search). The differential factor between docking pose predictions is, therefore, most represented by the scoring functions implemented in each software.

The GOLD CHEMPLP scoring function might match some aspects/characteristics of the FRED Chemgauss4 score function. As far as we may speculate, there are multiple linear potentials to model the van der Waals and repulsive terms included in CHEMPLP (in addition to hydrogen bonding terms [36]) that may work similarly to the shape interaction terms implemented in Chemgauss4, which are based on distances measured by van der Waals radii between atom models of heavy atoms [54]. This, but not only this, should indicate why both programs posed compound **1** inverted in relation to Glide and Autodock (of which their scoring functions are most known for their empirical-based features [55]). Nevertheless, we recognize that tracking down the factors that are involved in docking scoring functions that could provide different results in pose prediction should not be that obvious, and further tests will be necessary to clarify this.

Regardless, in order to clarify the most representative pose (supposed bioactive pose) of compound **1** in Pocket 7, i.e., what its possible mode of interaction in terms of orientation and conformation is, in Section 2.3, we also performed a study concerning surface, contour maps, and pharmacophore hotspot analysis using three complementary and independent software. These consensually indicated regions or chemical groups are most favorable in the places compatible with the compound **1** pose predicted by Glide docking. The superposition (see Figure 5) of **1** in close contact to residues that constitute allosteric Cavity 7 in GSK-3β was well-suited and showed coherent ligand–protein interactions, and, thus, we had one more reasonable argument to reinforce such a binding pose prediction.

In fact, we emphasize that three key interactions were depicted by this potential pose of **1** (obtained by Glide) within GSK-3β allosteric Cavity 7: the hydrogen bond between C=O of its carbohydrazide group and the N-H of the Ser236 backbone; cation-π (or electrostatic) interaction between its quinolone moiety and Arg209; and hydrophobic interactions between its aliphatic chain and hydrophobic regions Thr330, Pro331, and Arg328. This has also been previously described elsewhere [28] and corroborated by our contour map evaluation.

Regarding docking validation, we recognize that in previous docking validation (Section 2.4), a small set of compounds was used as allosteric inhibitors of GSK-3β; as they all came from the same series of 40 analogs with high structural similarity, it is likely that the results provided are limited. Therefore, it would be preferable to use compounds with greater structural diversity to validate the docking protocols and evaluate their efficiencies in proposing docking poses for different molecules towards GSK-3β allosteric Cavity 7.

In this way, in Section 2.5, we expanded the validation dataset in the docking assessment to include other compounds that have been found in the literature. Other works retrieved from the literature [24,25,26] revealed potential allosteric inhibitors, proved by their non-ATP-competitive inhibition at the GSK-3β catalytic site, and inferred how these compounds could bind to allosteric Cavity 7 by means of experiments or docking simulations (see Section 4.4).

Furthermore, we also performed similar validation procedures to ROCS queries, as showed in Section 2.6. For this task, the best-ranked poses of **1**, obtained by each docking software, were evaluated with respect to their use as a reference/query to run ROCS [45]. This software, which is based on shape similarity, requires a reliable pose (in terms of conformation) to perform a comparative search in the databases of compounds and rank them according to ROCSTC values.

For both validations (Table 2 and Figure 6), one should realize that the use of the expanded set of 88 compounds increases the structural diversity of the active/inactive compounds in relation to the series of 40 compounds used previously, also increasing the sample space of molecules and the respective domains of applicability for both docking protocols/programs and queries in ROCS. Thus, the validation procedure becomes more difficult, in which a more robust performance of each software evaluated will be required so that they present themselves as reliable.

In some cases, there was a decrease in the AUC values when considering decoys—which should further expand the structural diversity of the set—for example, for GOLD and Glide in docking validation (see Table 2). This may be attributed to the fact that there are trivial differences between the assets and the decoys generated or even due to the fact that they have not very dissimilar physicochemical properties.

Additionally, considering the high flexibility of molecule **1** (14 rotatable bonds) and its analogs, it is expected that among the 300 conformers for the inactive analogs of **1** (generated using OMEGA in a previous step to ROCS overlays), many conformation options will be generated, in which there will be a possible conformation with a high overlay, with **1** used as query, consequently resulting in a high ROCSTC value.

MD simulations were carried out to compare the stabilities of each potential complex formed between compound **1** in its two predicted poses by Glide and GOLD within allosteric Pocket 7 of GSK-3β. In general, results were equivalent, with slight indications that the Glide pose could form a more stable complex, indicated by its obtained RMSD values along the MD trajectory (see Figure 8d,e). Analysis of the MD results was consistent and also allowed us to check the potential of **1** to inhibit such enzymes allosterically. Studies in this sense may be a path to further explore, for instance, the analysis of the behavior of virtual hits towards GSK-3β allosteric inhibition.

Thus, after the investigation using different in silico tools and considering that **c**ompound **1** is a non-ATP-competitive inhibitor that acts on an allosteric site (thus confirmed by in vitro assays [28]), the results point in a consensual manner to the fact that it is most likely that **1** acts in allosteric Cavity 7 of the GSK-3β enzyme. In addition, we emphasize that this is indicated by all studies carried out (in Section 2.1, Section 2.2, Section 2.3, Section 2.4, Section 2.5, Section 2.6, Section 2.7) and, moreover, corroborates previous findings [28]. Finally, the pose of **1** (in terms of conformation and orientation, i.e., the ‘supposedly bioactive pose’), obtained by docking software Glide and the corresponding protocol (Figure 7), is the most reliable and robust one for prospecting ligand-based studies and seeking novel chemical entities or scaffolds.

Application of all of our virtual hypotheses and proposals to reinforce the reliability of the binding site and the ‘supposedly bioactive pose’ of compound **1** as a GSK-3β allosteric modulator could be firstly validated by using such content to perform the search for new structures with prospective intention. We emphasize that with the term ‘supposedly bioactive pose’, we refer to the orientation (together with the conformation) that such a ligand must assume, in three-dimensional space, in a most energetically favorable accommodation within the pocket (and with intermolecular interactions consistent with amino acid residues). This supposedly triggers a modulation in the activity of the enzyme, subsequently igniting other biochemical processes that will lead to an (un)expected biological effect/response.

Of course, objectively, obtaining such a crystallographic complex would be ultimately relevant. However, within our scope, performing virtual screening with the knowledge developed here and retrieving available existing chemicals to test if their inhibition potential is similar to the used reference is also a meaningful approach from our point of view.

Our robust VS pipeline was conducted in view of the filtering compounds most likely to present 3D shape similarity with the (query) docked pose of **1** since we filtered out many more compounds by this methodology—more than 13 million molecules were filtered out with ROCS, resulting in 15,000 molecules). In this sense, the ROCSTC values were pretty reasonable for the remaining compounds even after the application of other filters/methodologies (see Table 3).

Furthermore, we note the importance, in this study, of evaluating the ADME properties of GSK-3β allosteric inhibitors that will be selected by VS. This enzyme presents a wide range of functions in the body. In fact, although present in other compartments of the organism, this enzyme is overexpressed in the CNS of patients with neurodegenerative diseases, so that a given GSK-3β inhibitor to treat such diseases must have more affinity to the CNS than to the blood (which will distribute the drug to other compartments). This tendency can be controlled beforehand, in the previous steps of the design of inhibitors, by calculating and selecting the preferable values of the parameter logBB (Blood/Brain).

At the end of the VS pipeline, our visual inspection to pick compounds was performed in view of the docked poses (using Glide protocol), which showed valuable interactions (see Figure 9). This allowed us to carefully select compounds with promising activity towards the aimed target in addition to positively evaluated ADME/Tox profiles.

Quantum chemical studies also allowed us to check if our two selected virtual hits presented potential to interact in allosteric Pocket 7 of GSK-3β in a similar way as the reference compounds (already known as active allosteric modulators). Our calculations have provided interesting findings in this sense, especially with regard to HOMO and LUMO analysis.

Frontier molecular orbital is a relevant concept in chemistry, and it can be used extensively to describe the chemical reactivity behavior for a given molecule. HOMO represents the ability to donate electrons, being directly related to ionization potential, while LUMO represents the electron-accepting ability and its energy is associated with electronic affinity [48,49,50]. However, these molecular properties are global, and they can be related to chemical stability and reactivity; in this particular work, we stress that we made interpretations in terms of the ability of compounds to perform intermolecular interactions with the residues of the GSK-3β allosteric pocket.

Compounds **LCQFGS01** and **LCQFGS02** have shown energetic features, calculated by quantum chemical software, that agree with docking propositions, thus indicating their potential to allosterically inhibit GSK-3β by equivalent interactions in the same studied allosteric pocket.

In this way, all computational methodologies applied in this work will be validated by future experimental validation. Our two virtual hits exposed here, along with further hits obtained by VS as potential GSK-3β allosteric modulators, will be evaluated by in vitro enzymatic assays, with additional confirmation of the corresponding mechanism of action.

## 4. Materials and Methods

### 4.1. Pocket Detectors/Predictors

We employed fpocket [30], SuperStar [31], metaPocket [32], Sitemap [33], and PARS [34] to detect and predict the pockets present on the GSK-3β surface. These software usually exert pocket detection algorithms that allow the visualization of each cavity shape/volume along with corresponding calculated ‘scores’ values, according to the potential of the cavity to interact with potential ligands and/or drugs. We highly recommend that available details of each software in the corresponding references be checked in order to understand their particularities.

It is worth mentioning that we ran all software using a GSK-3β structure (PDB ID 1PYX) as input, and, when the software did not provide a default preprocessing workflow, the referred structure was treated by adding hydrogens, removing water molecules and other ligands/cofactors from the complex, and excluding the B subunit/chain from the protein structure using Maestro software [56].

### 4.2. Docking Simulations

Regarding the main ligand employed in docking simulations, i.e., compound **1**, this was prepared by the procedure established as default for molecules in this work, except when mentioned. Such procedure consisted of drawing its structure in the software ChemDraw [57] and copying it in SMILES format; this was then imported into Ligprep [58], which was run using an MMFF force field, calculating partial charges for atoms and generating possible ionization states by Epik at pH = 7.0 ± 1.0. Finally, the resulting file was exported in mol2 format for use. It is worth mentioning that this procedure resulted in a tautomeric form of **1**, in agreement with previous studies on minimum energy tautomeric predominance, as published elsewhere [28].

In advance of performing docking simulations, the protein structure was prepared by a procedure established as default in this work. Such procedure consisted of initially importing the Protein Data Bank (PDB, https://www.rcsb.org/, accessed on 18 May 2021) structure of the enzyme GSK-3β (PDB code 1PYX) into Protein Preparation Wizard software [59,60]. Then, its preprocessing was done by checking the following functions: assignment of bond orders using the CCD (Chemical Component Dictionary) database [61], addition of hydrogens, generation of disulfide bonds, use of Prime to fill missing loops and side chains, and removal of water molecules; only monomer A was kept, excluding monomer B, as well as ligands, cofactors, and metals; heavy atoms were converged and minimized to 0.30 Å of RMSD using the OPLS3 force field.

Each allosteric pocket investigated by docking methodologies in this work was defined by the following centroids (in terms of spatial coordinates): Pocket 4 (x = 30.09, y = −3.98, z = 30.83), Pocket 5 (x = 46.11, y = 20.74, z = 31.39), Pocket 6 (x = 40.28, y = 10.02, z = 46.49), and Pocket 7 (x = 11.78, y = 13.15, z = 38.33). In order to facilitate interpretation, note that these sequential enumeration of pockets corresponds to previous depiction in literature [21].

Preliminary docking simulations (results in Section 2.2) were carried out using default settings in each of the 4 four docking software. Further details of docking protocol refinement are shown in Section 4.5. Next, we only cite a few particularities from each default setting in each of the four docking software.

In GOLD, scoring function CHEMPLP and a sphere radius of 10 Å (centered at the centroid of each cavity) were used. In Glide, scoring function extra precision (XP) and grids of 10 × 10 × 10 Å (centered in each centroid) were used. In Autodock, default settings were used along with grids of 40 × 40 × 40 Å, centered in each centroid. In FRED, first, we used spruce4docking [62] to process the *apo* structure of GSK-3β (1PYX.pdb; previously prepared as described above) in order to generate ‘receptors’ for each pocket and thus indicate a representative residue for each pocket. Then, compound **1** was processed by OMEGA [47] to generate 300 conformers and submitted to docking run in (default) Standard mode.

### 4.3. Generation of Contour/Surface Maps

We employed Superstar [31], GRID [43], and Sitemap [33] to generate contour and surface maps using the GSK-3β structure (PDB ID 1PYX). Such protein structure was prepared as described in Section 4.1 in advance of running the software.

Superstar uses the empirical method to calculate maps that represent the propensity of a functional group (probe group) to bind at different positions around a protein-binding site. The software uses data from the Cambridge Structural Database (CSD) and the Protein Data Bank (PDB), using the IsoStar knowledge base as an intermediary [31].

GRID allows us to detect energetically favorable binding sites for functional groups on macromolecules by employing molecular interaction fields (MIFs) and different chemical probes. Its method is based on ‘grid points’ that are superimposed on the given protein, and the potential energy of each probe is then calculated using a predetermined chemical group The most important probes include water, the methyl group, amine, nitrogen, carboxy oxygen, and hydroxyl [43].

Sitemap performs contour maps calculation by identifying the hydrophobic, hydrogen-bond donor, the hydrogen-bond acceptor, and metal-binding regions on the protein using a grid of ‘site points’. For hydrophobic and hydrophilic character, the average of van der Waals interaction energy (over the original site and extended points of the probe–receptor) is computed; for the properties of donating/accepting hydrogen bonds, the algorithm is able to calculate and suggest which regions are preferred [33,63].

### 4.4. Dataset Compilation

As a starting point, we used the known set of 40 GSK-3β allosteric inhibitors (analogs of compound **1**) synthesized, with IC_50_ values measured, as reported [28].

In a preliminary search of the literature, we checked that there were not too many compounds reported specifically as GSK-3β allosteric inhibitors despite a considerable number of orthosteric inhibitors of the enzyme. Furthermore, lots of inhibitors have been previously described as non-ATP-competitive; however, a lack of data concerning which allosteric pocket should host such inhibitors does not allow researchers to use these data.

Exhaustive mining for more compounds was carried out, and we ended up finding more than 48 compounds that could be reliably considered, as shown in Table S1 (with references), totaling a compiled dataset of 88 GSK-3β allosteric inhibitors that should act in allosteric Pocket 7.

### 4.5. Refinement of Docking Protocols

Default settings (as procedures described in Section 4.2) were applied to previously prepared protein structures of GSK-3β (PDB 1PYX) and ligands (compound **1** plus further compounds of the dataset).

For all the software used here, i.e., GOLD, Glide, Autodock, and FRED, the results were programmed to generate 10 docking poses for each molecule; in some cases, fewer poses were generated (according to the energy cutoff established by each software). The poses obtained for each molecule were visually inspected and, thus, separated into ‘clusters’ (sets/families) of apparently better-overlapping poses that represented more homogeneous/significant poses. Among the family with the most significant poses, the one with the best score value was selected as the representative pose. When the results of the poses were very dispersed (low homogeneity between the poses), the pose with the best score was simply selected as the representative one for that molecule.

Constraints were not used in any of the software. In addition, different centroids (x, y, and z coordinates) were evaluated in relation to the distance between the main residues that constitute Cavity 7. Additionally, for GOLD and Glide, changes were also evaluated in relation to the flexibility of the side chains of the residues Arg209 and Ser236.

The following parameters were systematically varied in GOLD: sphere size spanning from 7 to 12 Å; CHEMPLP, ChemScore, and GoldScore score functions; population size and operations in ‘GA settings’; and allowing (or not) the inversion of N (amide) during the generation of conformers. Final docking protocol selected in GOLD was: centroid of x = 11.78, y = 13.15, z = 38.33; 10 Å sphere radius; 4 rotamers allowed for Ser236; the CHEMPLP scoring function.

In Glide: length of each grid side, varying from 10 to 20 Å; SP and XP score functions, allowing (or not) the inversion of N (amide) during the generation of conformers. Final docking protocol selected in Glide was: the same centroid as GOLD; the XP scoring function; not allowing N inversion; keeping the Ser236 residue flexible.

In Autodock, besides default settings, we also tried to run it as described in the literature [21]. The final docking protocol selected in Autodock was: centroid x = 10.508, y = 13.154, z = 37.338; grid of 40 × 40 × 38 Å with a spacing of 0.375 Å; Lamarckian GA (200 number of GA runs, 200 population size, 2,500,000 max number of evals); Ser236 flexible.

In FRED, we ran docking simulations in Standard, High, and Low resolutions (https://docs.eyesopen.com/applications/oedocking/fred/fred_opt_params.html#cmdoption-fred-dock_resolution, accessed on 18 May 2021). Final docking protocol selected in FRED was: indication of Ser236 to pick Pocket 7 in receptor generation by spruce4docking, then running docking in Standard resolution.

### 4.6. Validation of Docking Protocols

In order to validate each docking protocol developed for each docking software, we applied a validation procedure that consisted of obtaining docking scores (for each best pose) for the dataset of 88 compounds, including and not including additional DUD-E [44] decoys that were generated.

It is worth noting that previous, simple docking validation (Section 2.4) was carried out using only the small dataset of 40 known GSK-3β allosteric inhibitors (13 inactives against 27 actives) retrieved from the literature [28].

Decoys are potentially inactive structures, computationally generated from the input of active compounds on the webserver and the DUD-E database, with a view to presenting similar physicochemical properties but different chemical structures and topologies. These decoys were generated at http://dude.docking.org/generate (accessed on 18 May 2021) using the structures of 37 actives, leading to the generation of 50 decoys per active compound, i.e., ca. 1850 decoys. Thus, the decoys and 51 inactive compounds (retrieved from the 88-compounds dataset) were summed up and used against 37 known actives (also from the 88-compounds dataset), obtaining a classification dataset totaling ca. 1901 ‘inactives’ against 37 actives.

In this way, validation procedures were applied to the GOLD, Glide, Autodock, and FRED docking software, considering the respective score values, ranked (sorted from best to worst) in accordance with the corresponding binary codes, and indicating the activity/inactivity of the respective compounds, that is, 1 for active and 0 for inactive. The ROC curves were constructed using the Screening Explorer webserver (http://stats.drugdesign.fr/, accessed on 18 May 2021) [64] and were evaluated by the respective AUC values.

Validations through the analysis of ROC curves allow us to evaluate the capacity of a given methodology/model to correctly classify active compounds (i.e., compounds with known biological activity demonstrated by experimental results) within a set containing inactive compounds. In general, compounds are classified with respect to true/false positive/negative ratios and are represented in terms of sensitivity and specificity. Thus, for the construction of the ROC curves, sensitivity (*y*-axis) is correlated in the function of 1-specificity (*x*-axis), while sensitivity is defined by the ratio of true positives and the 1-specificity by the ratio of false-positives classified by the model. It is possible to evaluate ROC curves by the respective values of AUC, with values close to 1.0 indicating ideality and better performance of the model, while the value of 0.5 indicates a random classification by the model [65,66].

### 4.7. Shape Similarity and Query Validation

The best docking pose of compound **1** that was obtained by the best-chosen protocol developed for each software was validated to check its potential use as a query in a 3D shape similarity search using the software vROCS. Please note that we used vROCS, an alternative version to ROCS, which is generally preferred in validation studies, facilitating ROC-AUC curve analysis; it operates in an identical way as ROCS but allows graphic visualization.

Moreover, in addition to the poses of the 4 docking software, the minimal energy conformation for compound **1**, generated by OMEGA, was used as a pose, which is a strategy also recommended by the ROCS developers for the use, validation, and selection of queries [54,67].

The database of active compounds consisted of 37 molecules, and the database of inactive compounds consisted of either 51 molecules from the compiled dataset (see Table S1) and/or ca. 1901 ‘inactives’ when decoys were included (see Section 2.5 and Section 4.6). All databases were previously submitted to 300 conformer generation using OMEGA [47].

To classify/rank the molecules, the respective values of ROCSTC resulting from vROCS were considered. Such ROCSTC values vary from 0 to 2, with values closer to 2 showing a high overlap in terms of shape (ShapeTanimoto) and chemical characteristics (ColorTanimoto) between a given conformation of a molecule and the query used. From the generated results, the ROC curves were then plotted and the corresponding AUC values were obtained in a similar way to that described in Section 4.6.

### 4.8. Molecular Dynamics Studies

MD studies were conducted using high-performance computing (HPC) from *Centro de Computação de Alto Desempenho* at the University of São Paulo (USP). For MD simulations, we used GROMACS 2019.3 software [68,69] with the Charmm36 force field [70], and ligands were parameterized by CGenFF [69,71]. We obtained the initial coordinates to conduct the MD simulations from the docking poses of the ligands obtained by Glide and GOLD (as described in Section 4.2 and Section 4.5).

We placed the GSK-3β (PDB ID 1PYX) protein in a cubic box, with vectors 9.36 × 9.36 × 9.36 nm, and solvated it with 24,715 water molecules of TIP3P [72]. To neutralize charges in this system, we added 6 ions of Cl^−^ [69,73]. The total simulation time was 100 ns, with an integration time of 2 fs. To minimize the system, we used the Steepest Descent method to avoid unfavorable contacts between atoms, and we archived the convergence at potential energy below 500 kJ/mol.nm [73]. After that, we used the NVT and NPT sets for the balance/equilibrium runs, keeping the pressure constant at 1 bar through the Berendsen barostat and a temperature of 300 K, with time couplings of 2.0 and 0.1 ps, respectively [69,73]. Finally, we performed the structural analysis on the trajectory production, following the same protocol recently published [73].

### 4.9. Virtual Screening Campaign

We conducted a VS campaign, mostly represented by a ligand-based approach, using 3D shape similarity with the software ROCS. Table S2 shows the filtering of compounds.

Three databases were used in this work: Chembridge CNS [74], eMolecules [62], and Princeton [75]. Together, they sum ca. 13,000,000 molecules that are commercially available. These were prepared by application of FILTER [47], with default parameters and the following additional filter settings: maximum of 2 chiral centers, 5 ring systems, 20 atoms by ring system, 16 rotational bonds, and 55 rigid bonds. The use of FILTER is highly recommended [54] to eliminate unwanted/useless/unviable compounds in a molecular modeling pipeline—in advance of conformer generation and processing by OMEGA—mainly with respect to molecules bearing too many rotatable bonds (high flexibility) and rings (especially flexible ones). In sequence, OMEGA [47,76] was used to generate 300 conformers per molecule, considering the default parameters, except for the energy window set to 9.0 kcal/mol and RMSD adjusted to 0.6 Å (adaptations of our research group [77]). As the ROCS and EON software require conformational diversity between the structures of the query compound and other database molecules to perform overlap by shape and electrostatics, respectively, this processing step to generate the conformers using OMEGA is essential during the preparation of databases.

In ROCS [45,78], we used our predicted bioactive pose of compound **1** as a query and applied it to 3 selected and prepared databases. Hence, we filtered databases with ROCS, retrieving 5000 top-ranked molecules according to corresponding ROCSTC (shape + color) indices; moreover, screening was carried out through 3D electrostatic similarity using EON [79], selecting the 1000 top-ranked EONTC indices from each database.

In sequence, compounds were submitted to pharmacokinetic and toxicological (ADME/Tox) predictions using QikProp and DEREK, respectively. QikProp criteria to filter the most promising compounds were established considering CNS drug-likeness reports [80,81,82] and adapted by us, as follows: MW ≤ 360, PSA ≤ 90 Å^2^, (QP)logP_ow_ = −2.0 − 6.5; (QP)logBB > −0,5; heteroatoms ≤21; human oral absorption > 80%; (QP)P_Caco_ > 500 nm/s; (QP)P_MDCK_ > 500 nm/s. DEREK toxicity endpoints (carcinogenicity, genotoxicity, cardiotoxicity, hepatotoxicity, neurotoxicity, among others, for both mammals and bacteria) were predicted and rejected when respective alerts were fired as plausible, probable, or certain.

A final applied filter consisted of our previously developed and reliable docking protocol (using Glide) in order to evaluate the ability of survival compounds to establish consistent intermolecular interactions within the allosteric site of GSK-3β. Furthermore, visual inspection of their structures was narrowly conducted to select the most promising compounds with structural diversity.

### 4.10. Quantum Chemical Calculations

We performed quantum chemical calculations using GaussView 6.0 [83] and Gaussian 09 [84] software. In order to evaluate the energy of each compound—ensuring that their corresponding original docking poses (conformation + orientation) were kept—we employed two different methods of B3LYP hybrid density function theory (DFT) and the respective 6-311+G(d,p) and 6-311++G(2d,2p) basis sets [85,86,87]. In advance of choosing which basis set would be applied, we selected the one that presented greater stability according to the release of formation energy (see Table S3).

Following the procedure previously reported [52,88], we calculated the highest occupied molecular orbital (HOMO), the lowest unoccupied molecular orbital (LUMO), the GAP between LUMO and HOMO, the ionization potential (IP), and the spin density.

The topology of the frontier orbitals, HOMO and LUMO, was visualized with Gaussview software [83] and the ionization potential (IP) for each compound was calculated as the energy difference between a neutral molecule and the respective cation-free radical, i.e., following the equation *IP* = *E_molec_*^⦁+^ − *E_molec_*.

In this step of the work, we selected five compounds to investigate the relationship between their quantum chemical features and their potential to perform intermolecular interactions (within allosteric Pocket 7 of GSK-3β). These five selected compounds correspond to compounds **1**, **18**, and **24** (as three reference compounds from the literature [28] and also the most active ones (lowest values of IC_50_) towards GSK-3β allosteric inhibition; see Table S1), along with compounds **LCQFGS01** and **LCQFGS02** (as two potential hits obtained by our VS campaign). The idea was to compare the ability of these two virtual hits to perform inhibition (inhibit GSK-3β allosterically) in a similar manner to those three reference compounds in terms of the results observed from such quantum chemical calculations.

## 5. Conclusions

GSK-3β is undoubtedly a relevant protein kinase that is associated with multiple pathways of neurodegenerative diseases, thus representing a promising therapeutic target addressed to drug candidates in this context. Despite the fact that previous findings have suggested the advantageous strategy of inhibiting this enzyme kinase by allosteric modulators, mainly in view of their lowest chance of causing side effects, there are still some caveats regarding such a mechanism. Therefore, we performed an extensive in silico study, considering the main representative GSK-3β allosteric modulator, in order to revisit and reinforce the findings of which allosteric pocket this should bind as well as which pose interacts with the enzyme.

Methodologies ranged from cavity detection, ligand 3D shape analysis and docking (with validation of corresponding protocols/queries), and MD, and the results were consistent enough to provide new structural data, expanding the knowledge of GSK-3β allosteric inhibition. In order to apply the data gathered from our investigations, a 3D shape similarity VS campaign was conducted to validate our studies as well as increase the chemical and biological diversities of the compounds for this purpose. From this, we present two potential hits that succeeded in the ADME/Tox desired profile for CNS therapeutics and also in docking studies and quantum chemical analysis. Both these potential hits, plus further virtual hits that were obtained, will be tested by experimental in vitro assays in order to confirm their promising abilities to act as GSK-3β allosteric modulators.

## Figures and Tables

**Table 1:** Score values obtained using docking software GOLD, Glide, Autodock, and FRED for compound **1** against the four allosteric cavities (4, 5, 6, and 7) of GSK-3β.

GOLD-CHEMPLP				Glide-XP (Kcal/Mol)				Autodock-Binding Energy (Kcal/Mol)				FRED-Chemgauss4			
Cavity				Cavity				Cavity				Cavity			
4	5	6	7	4	5	6	7	4	5	6	7	4	5	6	7
54.88	71.20	56.07	88.36	−4.47	−5.85	−2.92	−6.13	−2.45	−4.12	−3.32	−6.98	−3.99	−3.46	−4.36	−5.53

**Table 2:** AUC values obtained in corresponding ROC curves plotted for docking and shape similarity (vROCS) validations, where queries refer to poses of compound **1** obtained by the corresponding docking software and applied to the dataset of 88 compounds, with and without decoys.

Docking Validation	88 Compounds	88 Compounds + *Decoys*	vROCS Validation	88 Compounds	88 Compounds + *Decoys*
GOLD	** **0.843** **	** **0.705** **	GOLD query	** **0.760** **	0.745
Glide	** **0.840** **	** **0.685** **	Glide query	** **0.744** **	** **0.756** **
Autodock	0.328	0.376	Autodock query	0.695	0.749
FRED	0.350	0.695	FRED query	0.745	** **0.758** **
			OMEGA query	0.684	0.684

In green: software that corroborate pose prediction studies (cavity detection and surface mapping); in red: software that do not corroborate; in bold: highest AUC values for corresponding green/red group in each situation. See also Table S4.

**Table 3:** Comparison of score values obtained by ROCS, EON, and Glide docking as well as ADME/Tox properties for compounds **1**, **LCQFGS01**, and **LCQFGS02**.

Compound	ROCS TC	EON TC	Glide XPscore	MW	PSA	^(QP)^logP_o/w_	^(QP)^logBB	HOA%	^(QP)^P_Caco_	^(QP)^P_MDCK_
1	2.000	2.000	−6.13	429.55	118.58	5.84	−2.04	94.2	375.19	171.46
LCQFGS01	0.884	0.720	−5.919	312.81	48.79	5.13	−0.42	100	2782.34	4074.68
LCQFGS02	0.917	0.672	−5.622	269.34	57.19	3.01	−0.35	100	1421.35	1121.06

**Table 4:** Calculated quantum chemical properties for the five selected compounds using DFT/B3LYP/6-311++G(2d,2p).

Compound	HOMO (eV)	LUMO (eV)	GAP *	IP (Kcal.mol^−1^)
1	−6.42	−1.94	4.48	181.06
18	−6.19	−1.79	4.40	174.75
24	−6.46	−1.86	4.60	182.23
LCQFGS01	−6.59	−1.77	4.82	192.97
LCQFGS02	−6.10	−1.03	5.07	177.12

* GAP = |E_LUMO_ − E_HOMO_|.

## Data Availability

The data presented in this study are available within this article and respective Supplementary Materials.

## Supplementary Materials

The following are available online at https://www.mdpi.com/article/10.3390/ijms22158252/s1. References [24,25,26,28] are cited in the Supplementary Materials.

## Abbreviations

AD	Alzheimer’s disease
ADME/Tox	Absorption, distribution, metabolism, and excretion/toxicity
APP	Amyloid precursor protein
ATP	Adenosine triphosphate
AUC	Area under the curve
B3LYP	Becke, 3-parameter, Lee–Yang–Parr
CNS	Central nervous system
DFT	Density function theory
EONTC	EON TanimotoCombo indices
FMO	Frontier molecular orbital
GSK-3β	Glycogen synthase kinase 3 beta
HOA%	Human oral absorption in %
HOMO	Highest occupied molecular orbital
IP	Ionization potential
LBVS	Ligand-based virtual screening
LUMO	Lowest unoccupied molecular orbital
MAPT	Microtubule-associated protein tau
MD	Molecular dynamics
MIFs	Molecular interaction fields
MW	Molecular weight
NFTs	Neurofibrillary tangles
PD	Parkinson’s disease
PDB	Protein Data Bank
PSA	Polar surface area
(QP)logBB	Logarithm of blood-brain barrier predicted by QikProp
(QP)logPo/w	Logarithm of partition coefficient in 1-octanol/water predicted by QikProp
(QP)PCaco	Permeability across Caco-2 cells predicted by QikProp
(QP)PMDCK	Permeability across Madin-Darby Canine Kidney cells predicted by QikProp
Rg	Radius of gyrate
RMSD	Root-mean-square deviation
RMSF	Root-mean-square fluctuation
ROC	Receiver operating characteristic
ROCSTC	ROCS TanimotoCombo indices
SASA	Solvent-accessible surface area
VS	Virtual screening
XPscore	Glide extra precision score values
