# Supplementary material for: Revisiting the Proposition of Binding Pockets and Bioactive Poses for GSK-3β Allosteric Modulators Addressed to Neurodegenerative Diseases
Source: Int J Mol Sci. 2021 Jul 31;22(15):8252. doi: 10.3390/ijms22158252 (PMC8348340; doi:10.3390/ijms22158252)
Supplement: Supplementary file 1 [file ijms-22-08252-s001.zip › ijms-1222907-supplementary.pdf]

Article - Supplementary Materials

Revisiting the Proposition of Binding Pockets and Bioactive Poses for GSK-3β Allosteric Modulators Addressed to Neurodegenerative Diseases

Guilherme M. Silva [1,*], Rosivaldo S. Borges [2,3,*], Kelton L. B. Santos [2,3], Leonardo B. Federico [4], Isaque A. G. Francischini [4], Suzane Q. Gomes [1], Mariana P. Barcelos [4], Rai C. Silva [1], Cleydson B. R. Santos [2,3,4] and Carlos H. T. P. Silva [1,2,4]

[1] Departamento de Química, Faculdade de Filosofia, Ciências e Letras de Ribeirão Preto, Universidade de São Paulo, Ribeirão Preto 14040-901, SP, Brazil; suzane.quintana@usp.br (S.Q.G.); raics@usp.br (R.C.S.); tomich@fcfrp.usp.br (C.H.T.P.S.)

[2] Programa de Pós-Graduação em Química Medicinal e Modelagem Molecular, Instituto de Ciências da Saúde, Universidade Federal do Pará, Belém 66075-110, PA, Brazil; keltonbelem@hotmail.com (K.L.B.S.); breno@unifap.br (C.B.R.S.)

[3] Laboratório de Modelagem em Química Computacional, Departamento de Ciências Biológicas e da Saúde, Universidade Federal do Amapá, Macapá 68902-280, AP, Brazil

[4] Laboratório Computacional de Química Farmacêutica, Faculdade de Ciências Farmacêuticas de Ribeirão Preto, Universidade de São Paulo, Av. do Café, s/n, Ribeirão Preto 14040-903, SP, Brazil; leonardobrunofederico@gmail.com (L.B.F.); isaque.francischini@usp.br (I.A.G.F.); mpbarcelos@usp.br (M.P.B.)

* Correspondence: silvagm@usp.br (G.M.S.); lqfmed@gmail.com (R.S.B.)

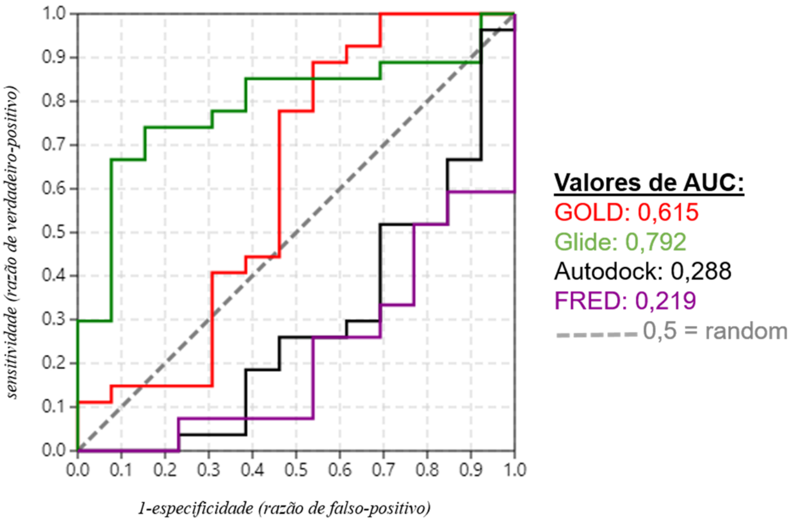

Figure S1. ROC curves obtained using only dataset of 40 compounds (**1** analogues) in order to perform docking validation.

Table S1. Dataset of 88 GSK-3β allosteric inhibitors compiled from literature[a, b, c, d] and used in this work to perform docking and vROCS-query validations, and corresponding IC_{50} (μM) values.

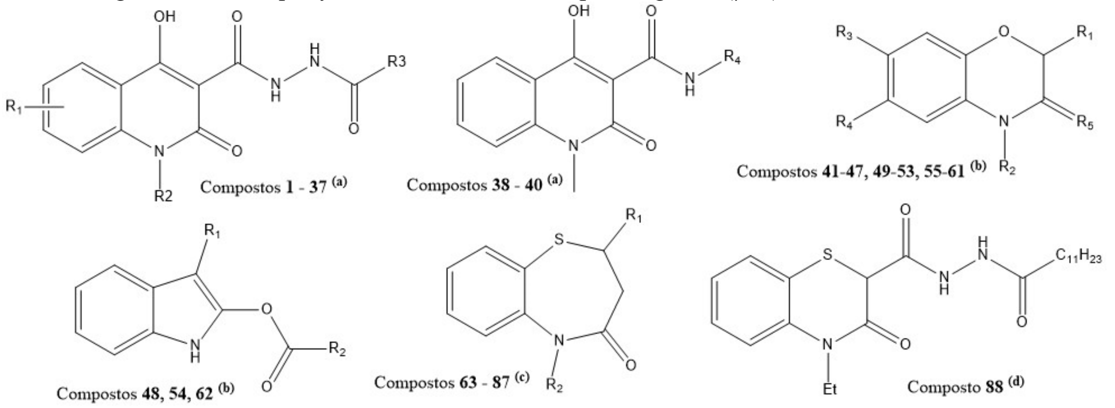

	R_1	R_2	R_3	R_4	R_5	IC_{50} (μM)		R_1	R_2	R_3	R_4	R_5	IC_{50} (μM)
**1**	H	Et	$C_{11}H_{23}$	-	-	2,80	**45**	1-naftil	4-OMe-bn	H	H	O	8,10
**2**	H	H	$C_{11}H_{23}$	-	-	4,50	**46**	1-naftil	4-F-bn	OCH2O	H	O	8,10
**3**	H	Me	$C_{11}H_{23}$	-	-	7,30	**47**	2-naftil	4-F-bn	H	H	O	8,70
**4**	H	Me	C_7H_{15}	-	-	8,70	**48**	1-naftil	Me	-	-	-	10,00
**5**	H	isoprenyl	$C_{11}H_{23}$	-	-	7,78	**49**	1-naftil	4-F-bn	1-morpholin	H	O	10,10
**6**	H	isoprenyl	C_7H_{15}	-	-	7,11	**50**	1-naftil	4-F-bn	F	H	O	21,20
**7**	H	CH_2-cyclopropyl	$C_{11}H_{23}$	-	-	5,25	**51**	1-naftil	Et	OCH_2O	H	O	25,60
**8**	H	Bn	$C_{11}H_{23}$	-	-	5,51	**52**	1-naftil	4-CH_3-bn	H	H	O	50,00
**9**	6-F	H	$C_{11}H_{23}$	-	-	5,80	**53**	phenyl	Bn	OCH_2O	H	O	50,00
**10**	6-Cl	H	$C_{11}H_{23}$	-	-	3,81	**54**	4-ch3-bn	Me	-	-	-	50,00
**11**	6-Cl	Me	$C_{11}H_{23}$	-	-	5,95	**55**	1-naftil	benzil	H	H	O	>50
**12**	6-Cl	Me	C_7H_{15}	-	-	8,48	**56**	1-naftil	4-Cl-bn	H	H	O	>50
**13**	6-Cl	Et	$C_{11}H_{23}$	-	-	3,18	**57**	1-naftil	4-NO_2-bn	H	H	O	>50
**14**	6-Cl	Et	C_7H_{15}	-	-	9,42	**58**	1-naftil	benzoyl	H	H	HH	>50
**15**	6-Br	H	$C_{11}H_{23}$	-	-	3,54	**59**	1-naftil	Me	OCH_2O	H	O	>50
**16**	6-Br	Me	$C_{11}H_{23}$	-	-	4,28	**60**	phenyl	Me	OCH_2O	H	O	>50
**17**	6-Br	Me	C_7H_{15}	-	-	6,28	**61**	phenyl	Et	OCH_2O	H	O	>50
**18**	6-Br	Et	$C_{11}H_{23}$	-	-	2,01	**62**	1-naftil	NEt2	-	-	-	>50
**19**	6-Br	Et	C_7H_{15}	-	-	7,34	**63**	2-Thienyl	Bn	-	-	-	47,50
**20**	7-Cl	H	$C_{11}H_{23}$	-	-	3,12	**64**	2-Furyl	Bn	-	-	-	77,20
**21**	7-Cl	H	C_7H_{15}	-	-	9,00	**65**	Ph	Bn	-	-	-	42,70
**22**	7-Cl	Me	$C_{11}H_{23}$	-	-	4,03	**66**	Ph	2-NO_2-Bn	-	-	-	25,00
**23**	7-Cl	Me	C_7H_{15}	-	-	6,66	**67**	Ph	2-Br-Bn	-	-	-	73,90
**24**	7-Cl	Et	$C_{11}H_{23}$	-	-	2,48	**68**	Ph	2-CH_3-Bn	-	-	-	76,10
**25**	7-Cl	Et	C_7H_{15}	-	-	4,83	**69**	Ph	4-CH_3O-Bn	-	-	-	73,70
**26**	7-Cl	Bn	$C_{11}H_{23}$	-	-	5,99	**70**	Ph	3-COOMe-Bn	-	-	-	27,80
**27**	7-Cl	Bn	C_7H_{15}	-	-	5,96	**71**	Ph	3-Cl-$PhCOCH_2$	-	-	-	37,80
**28**	H	H	C_7H_{15}	-	-	>20	**72**	PhCH2	2-NO_2-Bn	-	-	-	23,00
**29**	H	Me	Ph	-	-	>20	**73**	4-F-Ph	2-NO_2-Bn	-	-	-	81,50
**30**	H	Me	Me	-	-	>20	**74**	4-Cl-Ph	2-NO_2-Bn	-	-	-	71,30
**31**	H	Me	Bn	-	-	>20	**75**	4-Br-Ph	2-NO_2-Bn	-	-	-	67,80
**32**	H	Me	$(CH_2)2$-indol-3-yl	-	-	>20	**76**	2-Thienyl	Et	-	-	-	>100
**33**	H	Et	C_7H_{15}	-	-	>20	**77**	2-Thienyl	i-Pr	-	-	-	>100
**34**	H	Et	$(CH_2)2$-indol-3-yl	-	-	>20	**78**	2-Thienyl	n-Bu	-	-	-	>100
**35**	H	Et	$(CH_2)2$-(4-OH-Ph)	-	-	>20	**79**	2-Thienyl	Cyclohexthylmethyl	-	-	-	>100
**36**	H	isobutyl	$C_{11}H_{23}$	-	-	>20	**80**	2-Thienyl	Benzoyl	-	-	-	>100
**37**	6-F	H	C_7H_{15}	-	-	>20	**81**	H	Bn	-	-	-	>100
**38**	-	-	-	C_6H_{13}	-	>20	**82**	Me	Bn	-	-	-	>100
**39**	-	-	-	$CH_2CONHCH_2COOBn$	-	>20	**83**	3-Pyridyl	Bn	-	-	-	>100
**40**	-	-	-	NH-(CH2)2-CN	-	>20	**84**	Ph	2-CN-Bn	-	-	-	>100
**41**	1-naftil	SO_2-phenyl	H	H	HH	4,10	**85**	Ph	2-F-Bn	-	-	-	>100
**42**	1-naftil	4-F-bn	H	H	O	5,40	**86**	Ph	2-Cl-Bn	-	-	-	>100
**43**	1-naftil	bn	OCH_2O	H	O	6,40	**87**	Ph	3-COOH-Bn	-	-	-	>100
**44**	2-naftil	bn	OCH_2O	H	O	6,90	**88**	-	-	-	-	-	8,00

*In green compounds considered as actives, and in red compounds considered inactives, according to threshold of 20 μM of IC_{50} values. [a] PALOMO et al., 2017 [28]; [b] BROGI et al., 2017 [24]; [c] ZHANG et al., 2013 [25]; [d] ZHANG et al., 2014 [26].

Table S2. Number of compounds filtered in each step/filter performed in our virtal screening campaign.

Main methodology	Reference/Query	VS filter	Chembridge CNS	Princeton	eMolecules
ligand-based VS	Compound 1	-	53.306	511.340	12.607.334
		**ROCS (shape)**	5.000	5.000	5.000
		EON (electrostatic)	1.000	1.000	1.000
		QikProp (ADME)	536		
		DEREK (Tox)	332		
		Docking + Visual inspection	~ 30		

Table S3. Mean average values of released energy obtained for five compounds by each basis set.

**Compound**	**Energy released (kcal/mol)**	
	Basis set 1 (Hartree)[1]	Basis set 2 (Hartree)[2]
1	−1400.84	−1401.18
18	−3971.93	−3974.68
24	−1860.46	−1860.83
LCQFGS01	−1367.73	−1367.99
LCQFGS02	−860.69	−860.89
**Mean average**	**−1892.33**	**−1893.12**

[1] B3LYP/6-311+G(d,p); [2] B3LYP/6-311++G(2d,2p)

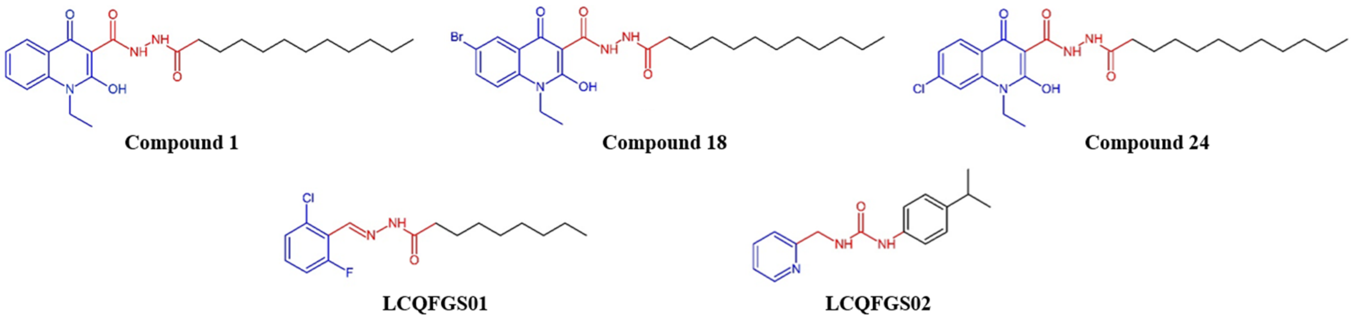

Figure S2. Representation of chemical structures of five selected compounds for quantum chemical studies. Blue and red portions respectively corresponds to moieties of quinolone and carbohydrazide for compounds **1**, **18**, **24**; halogenated benzene and methylenehydrazide for **LCQFGS01**; pyridine and carbamide for **LCQFGS02**.

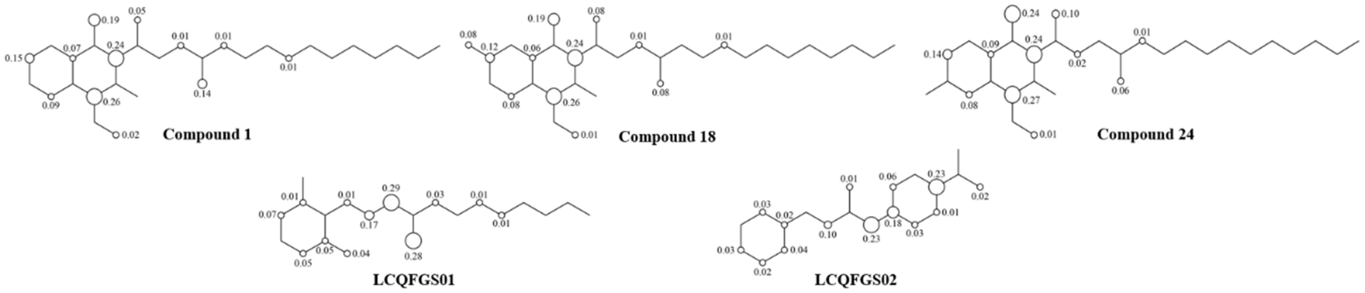

Figure S3. Spin density contributions of 5 compounds studied.

Table S4. AUC, TG and BEDROC values obtained in corresponding ROC curves ploted for docking and shape similarity (vROCS) validations, where queries refer to poses of compound **1** obtained by corresponding docking software, and applied to dataset of 88 compounds with and without decoys.

Docking validation	88 compounds			88 compounds + *decoys*			40 compounds*		
	AUC	TG	BEDROC	AUC	TG	BEDROC	AUC	TG	BEDROC
GOLD	0.843	0.474	0.729	0.705	0.310	0.223	0.615	0.169	0.848
Glide	0.840	0.455	0.985	0.685	0.240	0.177	0.792	0.381	0.993
Autodock	0.328	0.272	0.148	0.376	0.157	0.002	0.288	0.318	0.133
FRED	0.350	0.207	0.309	0.695	0.267	0.149	0.219	0.449	0.15

vROCS validation	88 compounds			88 compounds + *decoys*		
	AUC	TG	BEDROC	AUC	TG	BEDROC
GOLD *query*	0.760	0.496	0.947	0.745	0.583	0.666
Glide *query*	0.744	0.475	0.772	0.756	0.578	0.648
Autodock *query*	0.695	0.400	0.287	0.749	0.540	0.623
FRED *query*	0.745	0.500	0.932	0.758	0.580	0.668
OMEGA *query*	0.684	0.496	0.943	0.684	0.570	0.670

AUC: Area Under the Curve (calculated from respective ROC curves); TG: Total gains quantify the association between the variations of the scores and the detection of active compounds; BEDROC: Boltzmann Enhanced Discriminations of ROC quantify the early recognition of active compounds. All metrics retrieved from http://stats.drugdesign.fr/;

* Smaller dataset of 40 compounds used in preliminary docking validation (section 2.4 and Figure S1).
